# Consider hospice in end‐stage liver disease prognostic scale to open discussions regarding six‐month mortality

**DOI:** 10.1002/jgh3.12889

**Published:** 2023-03-20

**Authors:** Cristal Brown, Nazan Aksan, Andrew Joseph Muir

**Affiliations:** ^1^ Dell Medical School University of Texas at Austin Austin Texas USA; ^2^ Duke University School of Medicine Duke Clinical Research Institute Durham North Carolina USA

**Keywords:** cirrhosis, hospice, mortality, palliative care, prognosis

## Abstract

**Background and Aim:**

Hospice is underutilized in the management of patients with end‐stage liver disease and may improve the patient experience at the end of life. This study aims to create a novel prognostic scale to accurately predict 6‐month mortality to more comprehensively facilitate hospice referral.

**Methods:**

Sociodemographic, clinical, and laboratory variables associated with mortality from the United Network for Organ Sharing database were tested in univariate analysis followed by multivariate analyses with four predictor groups: Demographics, Diagnoses, Complexities, and Laboratory studies to develop the hospice in end‐stage liver disease prognostic scale (HELP) scale (70% sample, *N* = 13 516) followed with replication in a 30% (*N* = 5792) internal validation sample.

**Results:**

Only the predictor groups of Complexities and Laboratory studies met the *c*‐statistic threshold of 0.70 for inclusion in the multivariate analyses. Backward elimination in the final logistic regression and validated weighted transformation procedure resulted in: HELP scale = (functional status × 11) + (ascites × 3) + (SBP × 3) + (HE × 4) + (dialysis × 5) + (TIPS × −3) + (albumin × −3) + (MELD‐Na ≥ 21 × 20). HELP scale had a strong predictive value for six‐month mortality with Area under the Receiver Operating Curve (AUROC) 0.816 and replicated in the validation sample.

**Conclusion:**

HELP scale is a novel prognostic score utilizing the strength of model of end‐stage liver disease‐sodium (MELD‐Na), along with clinical factors, for a more nuanced assessment of six‐month mortality. This scale can provide an individualized approach in opening discussions of hospice referral and may be better accepted by patients and providers given its contextualization of important clinical factors.

## Graphical abstract

Current hospice criteria are underutilized for consideration of hospice referral. The Hospice in end‐stage liver disease prognostic scale is a novel prognostic score utilizing the prognostic strength of model of end‐stage liver disease‐sodium (MELD‐Na) with the addition of clinical factors for a more nuanced assessment of six‐month mortality. This scale can provide a greater individualized approach to aid providers in opening discussions of hospice referral and may be better accepted by patients and providers given its contextualization of important clinical factors in addition to the foundation of MELD‐Na.

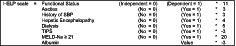

## Introduction

End‐stage liver disease (ESLD) has a growing impact on the American healthcare system related to increasing prevalence and deaths.^1^, ^2^, ^3^ The burden of liver disease is expected to continue increasing due to the aging population, increasing alcohol‐associated liver disease and unchecked obesity epidemic.^3^, ^4^ The literature consistently demonstrates that the high resource utilization incurred during the end of life (EOL) management of this patient population has not translated to improved quality of life (QOL).^5^, ^6^, ^7^ Our current model of EOL care for patients with ESLD is suboptimal and innovative methods are needed to improve the patient experience at the EOL.

Hospice is a team‐oriented approach to providing quality and compassionate care for patients with life‐limiting illness.^8^ Maintaining patient‐centered QOL in hospice management allows emphasis on domains of supportive care which are often lacking in the traditional EOL management of patients with ESLD.^7^, ^9^, ^10^ The benefits of hospice in the EOL management of patients with ESLD is poorly understood because it is currently underutilized.^11^, ^12^ ESLD does not fall within the top 15 principal diagnoses for hospice enrollment in Medicare decedents despite being the 11th leading cause of death in the United States.^13^, ^14^ Yet advanced cancer has demonstrated significant benefits using hospice to improve QOL during terminal illness and early data suggests that patients with chronic end‐organ illnesses such as ESLD may also confer similar benefits.^15^, ^16^ Understanding and addressing the barriers to hospice referral are vital to improving the appropriate utilization of this service to enhance the EOL experience of patients with ESLD.

An important barrier to hospice referral, for both patients and providers, is a poor understanding of mortality risk due to the uncertainty of the disease course.^17^, ^18^ Decompensated liver disease is characterized by a prolonged, undulating course that can make prognostication difficult.^19^ Providers must ascertain the likelihood of six‐month mortality as a condition of hospice referral and the prognostic uncertainty often leads to delayed hospice referrals for this patient population.^5^, ^20^

Acknowledgment of the underutilization of hospice at the EOL for decompensated cirrhosis is becoming increasingly recognized on a national level and has led to the publication of the first practice guidance for palliative care and symptom‐based management in decompensated cirrhosis.^21^ Included in this guidance are the current hospice enrollment criteria for advanced liver disease and the suggestion of the use of MELD ≥ 21 as a prognostic indicator for six‐month mortality.^22^, ^23^ While model of end‐stage liver disease‐sodium (MELD‐Na) has been found to be a useful predictor for six‐month mortality in patients with ESLD; it has not been significantly utilized in clinical practice to identify patients that may benefit from consideration of hospice referral.^24^, ^25^, ^26^

An explanation for the underutilization of MELD‐Na for hospice enrollment may be its purely objective formula. Recent literature demonstrates that assessment of clinical factors such as ascites, encephalopathy, and frailty provides a more comprehensive prognostic picture to allow providers to engage patients and caregivers in discussions of advance care planning and consideration of hospice.^19^ Physicians and patients may be uncomfortable delving into conversations regarding mortality and EOL based solely on lab values. A recent expert opinion regarding hospice care for ESLD in the United States concluded that the limitations of established prognostic measures, including MELD‐Na, signify a need for a new approach to data‐driven hospice criteria to assist the patient and provider in considering hospice referral despite the uncertain course of advanced liver disease.^11^

## Objective

Creation and validation of a novel prognostic scale for patients with ESLD incorporating sociodemographic, clinical, and laboratory variables to MELD‐Na ≥ 21 that can accurately predict six‐month mortality to assist patients and providers in considering hospice referral.

## Methods

### UNOS database

The United Network for Organ Sharing (UNOS) database was utilized as a large database of patients with decompensated cirrhosis providing sociodemographic, clinical, and laboratory data. This study was IRB‐exempt due to the use of human subjects' data obtain that is publicly available and de‐identified. Data usage agreement was signed and data from 1 October 1987 to 30 September 2019 was provided. Cross‐sectional, retrospective analysis was performed from UNOS data for the listing year 2015 and later based on the change in use to MELD‐Na in 2016.

### Sample

**Figure 1:**
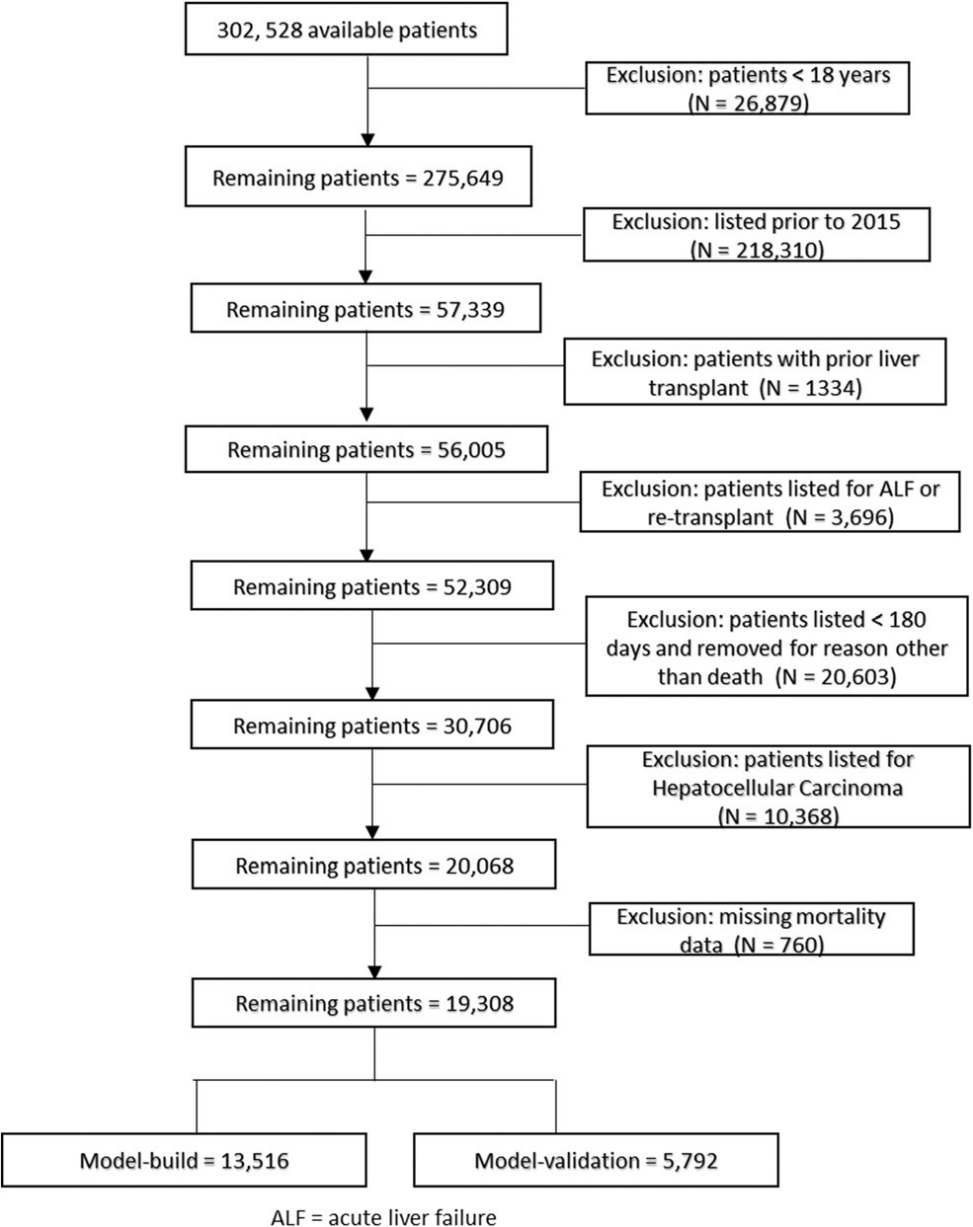
Flow chart of exclusion criteria and eligible subjects.

Inclusion criteria consist of patient age ≥ 18 years and carrying a diagnosis of cirrhosis. Patients with a diagnosis of hepatocellular carcinoma (HCC) were excluded based on extensive missing data and distinct clinical course from those patients listed for liver transplantation with decompensated cirrhosis. Exclusion criteria also include fulminant hepatic failure as an indication for transplantation, history of prior liver transplantation, and receipt of liver transplantation in less than 180 days on the waiting list. Figure 1 shows a flow chart for the selection of eligible patients. Among the eligible 20 068 patients, 760 patients had missing data on the timing of mortality (less than 180 days or later) and 19 308 patients remained for analysis.

### Primary outcome

Identification of clinically relevant variables from the UNOS wait list that are predictive of <180‐day mortality.

### Measures

Nineteen variables were identified from the 404 possible wait list variables available based on a systematic review of 118 studies examining prognostic indicators for survival in cirrhotic patients.^27^ These variables included demographic information, socioeconomic factors, etiology of liver disease, co‐morbid conditions, complications from portal hypertension, and laboratory studies.

### Analytic plan

Frequency distributions for categorical and continuous predictors and the outcome of 180‐day mortality were examined to determine potential sparseness issues. Given the number of potential predictors and distribution of the outcome, 70% of the cases were used for model‐building purposes and 30% for validation. The model‐build (*N* = 13 516) and model‐validation subsamples (*N* = 5792) were formed to ensure a similar proportion of survivors at 6‐months (82%) *versus* not (18%) in each random sample. Both univariate and multivariate analyses were conducted first in the model‐build subsample and tested in the validation subsample. The analytic steps that were followed to construct the final model in the model‐build sample and associated prognostic scale are described first. The procedures that were used to test model adequacy and evaluation of the prognostic scale in the validation sample are described second.

The following analytic steps were followed to arrive at the final model in the model‐build sample which was used to create the new prognostic score:

Step‐1: Bivariate logistic regressions for categorical and continuous predictors were used to predict the binary outcome of 180‐day mortality. The following variables were consolidated to construct clinically relevant categories for analysis:
  - *Education* was made binary as completion of 0–12 grade (HS or less) or beyond high school graduation
  - *Ethnicity* and *ethnicity categories* were combined to create **Minority status**. Minority status was made binary as minority and non‐minority. Non‐minority was defined as ethnicity category = white and ethnicity = non‐Hispanic.
  - **4‐category diagnoses** were created including viral hepatitis, nonalcoholic fatty liver, alcohol, and other diagnosis.
  - *Diabetes* reduced to 2‐category presence or absence of diabetes.
  - *Ascites* reduced to 2‐category presence or absence of ascites.
  - *Functional status* was consolidated to a binary variable comprised of total independence, or at least some dependence.
  - *Labs* included continuously measured albumin and MELD‐Na as a categorical variable with ≥21 as the cut‐off following recent practice guidance
  - *Any previous malignancy* was a 2‐category variable, note that HCC was an exclusion criterion.
Step‐2: The variables were grouped into the following four predictor sets:
  - *Demographics* (gender, age, education, BMI, and minority status)
  - *Diagnoses* (diabetes and four‐category diagnoses)
  - *Complexities* (ascites, prior abdominal surgery, functional status, portal vein thrombosis [PVT], spontaneous bacterial peritonitis [SBP], transjugular intrahepatic portosystemic shunt [TIPS], hemodialysis [HD], non‐hepatic malignancy, hepatic encephalopathy [HE]; each as a binary outcome as described in Step‐1).
  - *Laboratory studies* (albumin, MELD‐Na ≥ 21)
Step‐3: The grouped predictors in Step‐2 were each submitted to separate multivariate logistic regressions with backward elimination and the associated *c*‐statistics were computed. If the 95% confidence interval (CI) of the *c*‐statistic associated with a given multivariate logistic regression included values higher than 0.70 then the predictors retained in that multivariate regression were included in the final logistic regression.
Step‐4: The final logistic regression relied on backward elimination starting with all predictors that met the criterion to be retained in Step‐3.
Step‐5: The coefficients obtained from the final model obtained in Step‐4 were used to create a weighted prognostic scale in the model‐build sample and its *c*‐statistic was examined.^28^

To assess the adequacy of the multivariate model and prognostic scale score from Step‐5 in the validation sample, we adopted the following procedures: (a) assess whether the decisions made in the model‐build sample in Step‐3 replicated in the validation sample; (b) assess the similarity in the predictors and their coefficients in the final logistic regression that emerged in Step‐4 in the validation sample; (c) apply the weighted prognostic scale from the model‐build sample to the validation sample and evaluate the similarity of its *c*‐statistics to those in the build sample.

## Results

### Preliminary descriptive analyses for the whole sample

Descriptive statistics were obtained for continuous and categorical variables for the whole sample. Table 1 shows the means and standard deviations of the continuous predictors, frequency distributions for the categorical predictors, and missing data percentage. Missing data were generally low.

**Table 1:** Descriptive statistics for categorical and continuous variables for the whole sample

Predictors	Frequency *N* (%)	Mean	SD	Missing data, *N* (%)
Demographics				
Gender				
Male	11 248 (58.3)			0 (0.00)
Female	8060 (41.7)			
Age		55.16	10.82	0 (0.00)
Education^┼^				
HS less	8769 (45.4)			750 (3.9)
Beyond HS	9789 (50.7)			
BMI		29.01	6.16	37 (0.002)
Race				
White	13 ,847 (71.7)			0 (0.00)
African‐American	1285 (6.7)			
Asian	619 (3.2)			
Hispanic	3253 (16.8)			
Native American	180 (0.90)			
Pacific Islander	19 (0.10)			
Multi‐racial	105 (0.50)			
Ethnicity				
Hispanic	3282 (17.0)			0 (0.00)
Minority status^┼^				
Minority	5461 (28.3)			0 (0.00)
Non‐minority	13 ,847 (71.7)			
Diagnostic predictors				
Diagnosis^┼^				
Viral Hepatitis	2768 (14.3)			521 (2.7)
NASH	4462 (23.1)			
Alcohol	6530 (33.8)			
Other diagnosis	5027 (26.0)			
Diabetes^┼^	6,147 (31.8)			52 (0.003)
Complexities				
Ascites^┼^	14 ,832 (76.8)			0 (0.00)
Prior abdominal surgery	9235 (47.8)			328 (1.7)
Functional status^┼^				
Some ADL dependence	12 ,308 (70.9)			1943 (10.1)
PVT	1640 (8.50)			97 (0.005)
SBP	1653 (8.60)			130 (0.70)
TIPS	2113 (10.90)			398 (2.1)
Dialysis week prior to listing	1496 (7.70)			3 (<0.01)
History of prior malignancy (malignancy)	1503 (7.8)			0 (0.00)
Hepatic encephalopathy	11 983 (62.1)			0 (0.00)
Labs				
Child‐Pugh Score		8.04	1.63	1 (<0.01)
Albumin		3.14	0.64	1 (<0.01)
MELD‐Na		19.62	7.82	1 (<0.01)
MELD‐Na 21	7412 (38.4)			1 (<0.01)
Death relevant information^‡^ ^,^ ^§^				
Death within 180 days	3637 (18.8)			0 (0.00)
Median number of days to death	2102 (57.7)	40	75	1535 (43.3)
Cause of death: Multi‐organ failure	709 (19.5)			
Cause of death: Sepsis	236 (6.5)			
Cause of death: Cardiac arrest	149 (4.1)			
Cause of death: Other	692 (19.0)			
Cause of death: Unknown	316 (8.6)			

^┼^ Transformed variables used in the analysis as described in analytic plan step 1.

^‡^ Dates from listing to death were available for only 2102 patients and we are reporting median and interquartile range for this variable and percentages are relative to 3637 total deaths.

^§^ Cause of death information were only available for a subset of the patients, only the most common causes are listed in Table 1

ADL, activities of daily living; BMI, body mass index; HS, high school; NASH, non‐alcoholic steatohepatitis; PVT, portal vein thrombosis; SBP, spontaneous bacterial peritonitis; TIPS, transjugular intrahepatic portosystemic shunt.

## Model building/selection

### Bivariate analysis

Table 2 provides the odds ratio for 180‐day mortality and its 95% CI obtained from bivariate logistic regressions in the model‐build sample (*N* = 13 516). In addition to odds ratios, Table 2 provides the mean and its 95% CI for continuous variables among survivors and non‐survivors. All of the bivariate associations were in the expected direction.

**Table 2:** Bivariate analyses for categorical and continuous variables in the model build sample

	Odds ratio (95% CI) for 180‐day mortality	Died <180 days (*N* = 2910)	Survived at least 180 days (N = 12 537)
		Mean (95% CI)	Mean (95% CI)
Demographics			
Female	1.208 (1.107, 1.317)		
Age (years)	1.025 (1.02, 1.03)	57.3 (56.9, 57.7)	54.64 (54.4, 54.8)
Education	1.187 (1.086, 1.298)		
BMI^†^	1.004 (0.997, 1.011)	29.1 (28.9, 29.4)	28.9 (28.8, 29.1)
Minority	1.133 (1.031, 1.245)		
Diagnostic predictors			
Diagnosis at listing			
Viral	0.892 (0.770, 1.033)		
NASH	1.087 (0.961, 1.230)		
Alcohol	1.113 (0.995, 1.245)		
Other diagnosis	0.98 (0.934 1.02)		
Diabetes	1.042 (0.95, 1.143)		
Complexities			
Ascites	2.612 (2.301, 2.966)		
Prior abdominal surgery	1.055 (0.967, 1.151)		
Functional status some to total ADL dependence	4.606 (3.983, 5.326)		
PVT	1.08 (0.933, 1.26)		
SBP	2.01 (1.76, 2.30)		
TIPS	0.738 (0.635, 0.857)		
Dialysis week prior to listing	4.09 (3.60,4.67)		
Non‐hepatic malignancy	0.897 (0.76, 1.06)		
Hepatic encephalopathy	2.15 (1.95–2.38)		
Labs			
Albumin (g/dL)	0.61 (0.57, 0.65)	2.99 (2.96, 3.02)	3.19 (3.18, 3.20)
MELD‐Na > 21	9.40 (8.47–10.43)		

^†^ BMI reported to 3 decimal places to show its significance above OR 1.0 and retention in Demographics predictor set.

ADL, activities of daily living; BMI, body mass index; HCC, hepatocellular carcinoma; NASH, non‐alcoholic steatohepatitis; PVT, portal vein thrombosis; SBP, spontaneous bacterial peritonitis; TIPS, transjugular intrahepatic shunt.

### Multivariate analyses

The variables shown in Table 2 were grouped into predictor sets as described in the methods section. These predictor sets were submitted to separate logistic regressions with backward elimination. The final set of predictors that were retained in each of these regressions was used to obtain *c*‐statistics (area under the curve) to evaluate whether as a set of predictors, they were clinically promising with regard to six‐month mortality. We considered a set of predictors as potentially useful if the associated 95% CI for the *c*‐statistic contained values of 0.70 or higher. These values are provided in Table 3 along with the predictors that were retained. As a predictor set, neither diagnoses nor demographics met the 0.70 thresholds for inclusion consideration in the final logistic regression, described in Step‐2 of the methods. In addition, prior abdominal surgery, PVT, and malignancy indicators from the Complexities predictor set were eliminated from these multivariate logistic regressions. Hence, only the predictor sets associated with complexities and labs met the *c*‐statistic threshold of containing 0.70 in the 95% CI (listed in the last column of Table 3). The remaining predictor sets were dropped from further consideration.

**Table 3:** *c*‐statistic (AUC) and its 95% CI for predictor subsets, and variables retained in the final equation for the build sample

Predictor groupings	AUC, (95% CI)	Variables retained
Demographics	0.58 (0.568, 0.593)	None
Diagnoses	0.52 (0.507, 0.532)	None
Complexities	0.709 (0.698–0.721) 0.696 (0.677–0.714)	Ascites, functional status, SBP, TIPS, hepatic encephalopathy, dialysis week prior to listing
Labs	0.778 (0.769, 0.787)	Albumin, MELD‐Na ≥ 21

BMI, body mass index; SBP, spontaneous bacterial peritonitis; TIPS, transjugular intrahepatic portosystemic shunt.

### Final multivariate model

Using Step‐4 from the analytic process described in the methods section, the final logistic regression model used backward elimination on all of the predictors retained in predictor subsets, complexities, and labs, shown in Table 3. Table 4 provides the odds ratios and associated 95% CIs for each term from this multivariate regression. Using the procedures discussed in Mehta *et al*.^28^ a weighted prognostic scale using final model coefficients was formed. Each coefficient was multiplied by 10 and rounded up.^28^ Both the coefficients and the weights are shown in Table 4. The *c*‐statistic for the hospice in end‐stage liver disease prognostic (HELP) scale in the build sample was 0.816 (0.806, 0.826).

**Table 4:** Significant predictors in final logistic regression model with associated OR, 95% CI and assigned scale weight for the coefficient in the build sample

		Scale		95% CI		
	Coefficient	Weight	Odds Ratio	Lower Limit	Upper Limit	*P*‐value
Functional status	1.093	11	2.982	2.543	3.496	<0.001
Ascites	0.258	3	1.294	1.102	1.521	0.002
SBP	0.326	3	1.385	1.176	1.631	<0.001
Hepatic Encephalopathy	0.431	4	1.539	1.358	1.743	<0.001
Dialysis	0.544	5	1.722	1.473	2.014	<0.001
TIPS	−0.298	−3	0.742	0.622	0.886	0.001
Albumin	−0.245	−3	0.783	0.722	0.849	<0.001
MELD‐Na ≥ 21	1.969	20	7.160	6.342	8.083	<0.001

### Model evaluation in the validation sample

The final multivariate logistic regression in the validation sample was very similar to that shown in Table 4 for the build sample. The HELP score was computed in the validation sample using the weights derived from the model‐build sample. The *c*‐statistic and its 95% CI for this weighted score in the validation sample were 0.808 (0.792, 0.824). Table 5 provides the coefficients for the HELP scale for the entire sample.

**Table 5:** Final multivariate model with weighted prognostic score for the whole sample

HELP scale =	Functional status	(Independent = 0)	(Dependent = 1)	*11
	Ascites	(No = 0)	(Yes = 1)	*3
	History of SBP	(No = 0)	(Yes = 1)	*3
	Hepatic Encephalopathy	(No = 0)	(Yes = 1)	*4
	Dialysis	(No = 0)	(Yes = 1)	*5
	TIPS	(No = 0)	(Yes = 1)	*−3
	MELD‐Na ≥ 21	(No = 0)	(Yes = 1)	*20
	Albumin		Value	*−3

### Predictive power

Table 6 lists various cut‐off values on the HELP scale and the associated counts concerning true positive, true negative, false positive, false negative, positive predictive value (PPV), negative predictive value (NPV), in addition to the mean and 95% CI for the predicted probability of death from the multivariate logistic regression. Table 6 indicates that PPV and NPV, the values most meaningful to the patient perspective are maximized at values 28 and above on the HELP scale and that these values correspond to a greater than 50% probability of death in the following six‐month period.

**Table 6:** HELP scale cut‐off values and associated statistics: true negative, true positive, false positive, false negative counts, PPV, NPV, and predicted probability of death

					Pr(death within 180 days)		
HELP cut‐off	TN/TP	FP/FN	PPV	NPV	mean	LL	UL
28	12 274/1254	1257/1587	52.38	87.52	0.5	0.499	0.503
29	13 026/976	813/2104	55.53	85.61	0.524	0.522	0.527
30	13 030/765	582/2113	59	84.92	0.543	0.54	0.546
31	13 438/587	401/2493	60.36	83.82	0.562	0.559	0.565
32	13 457/434	263/2519	63.97	83.24	0.582	0.579	0.586
33	13 677/305	162/2775	66.2	82.57	0.603	0.599	0.606
34	13 692/214	99/2789	76.07	82.63	0.62	0.616	0.624
35	13 743/143	54/2872	73.72	81.87	0.639	0.634	0.643
36	13 808/85	31/2995	74.04	81.59	0.658	0.653	0.663

FN, false negative; FP, false positive; LL, lower limit of the 95% CI; NPV, negative predictive value; PPV, positive predictive value; Pr, predicted probability; TN, true negative; TP, true positive; UL, upper limit of the 95% CI.

## Discussion

This study uses the largest database of patients with decompensated cirrhosis in the United States to create and validate a prognostic scale for six‐month mortality, the HELP scale. The scale identifies six clinical variables, added to MELD‐Na ≥ 21 and albumin, that provide strong predictive value for six‐month mortality. This data‐driven approach provides a more holistic picture of a patient suffering from the complications of decompensated cirrhosis. Providers subjectively understand that the presence of multiple co‐morbidities and complications of portal hypertension increase cumulative mortality risk using the commonly referred “eye‐ball test” but this has not been well captured in prognostic scales for six‐month mortality to aid in decisions regarding the utility of hospice referral. The lack of inclusion of clinical factors may account for the limited use of established prognostic scoring systems as hospice criteria for referral. Our novel scoring system specifically targets six‐month mortality and incorporates clinical factors that can assist the provider in explaining the risk of death in a more individualized approach to engage patients in discussion regarding advance care planning and potentially hospice.

The HELP scale ranges from −12 (MELD‐Na < 21, without listed complications and albumin 4.0) to 49 (MELD‐Na ≥ 21, with all listed complications and undetectable albumin). A HELP scale score of 28 or higher carries a ≥50% probability of six‐month mortality and meets the requirements for hospice enrollment with likely death in six‐months if a typical disease course unfolded. Yet this scale also answers the call to use markers such as ascites, encephalopathy, and frailty to provide a more nuanced clinical risk assessment for mortality.^11^, ^19^

Patients with a MELD‐Na ≥ 21 and any level of functional dependence will meet the criteria for hospice discussion by the HELP scale which underscores the importance of functional status, even in the absence of major complications of portal hypertension for patients with a high MELD‐Na score. The HELP scale also demonstrates that a MELD‐Na ≥ 21 with only 1 listed complication of portal hypertension does not meet the threshold for opening a hospice discussion but the cumulative effect or two or more listed complications will generally meet the threshold of 28 and may serve as a trigger to providers to begin discussions regarding the utility of hospice referral.

There is a growing understanding of the importance of functional status in the prognosis of patients with ESLD.^29^ Our model supports an association between decreased functional status and increased mortality. There were <10% of patients meeting inclusion criteria with total dependence, defined as a Karnofsky Performance Score of 0–40, and 10% missing data for functional status. These factors necessitated the assessment of functional status as a binary variable of total independence or some level of dependence. This binary classification does not allow a more nuanced assessment of functional status and likely underestimates the importance of functional status in mortality. It is anticipated that functional status will have a greater impact when assessing patients in other samples that have an increased number of patients with severe disabilities.

In the final model, the use of TIPS was found to be protective. This has also been recently shown in a multicenter study evaluating post‐TIPS mortality for indications of refractory ascites, hepatic hydrothorax, and variceal hemorrhage.^30^ Our study, along with Boike *et al*. demonstrate that TIPS can be protective in certain populations despite its association with refractory symptoms of ESLD, signifying increased portal hypertension. This increased portal hypertension often correlates with increased mortality. In our population, patients are likely healthier than the general population of ESLD patients based on their listing for liver transplantation. Therefore, the use of TIPS likely serves as a temporizing measure to maintain appropriate clinical status for liver transplantation. Yet transplant candidacy is only one factor applicable in making appropriate patient selection for TIPS. Patients that are ineligible for liver transplant often benefit from the use of TIPS in the management of complications of portal hypertension and further investigation regarding its impact on six‐month mortality is warranted in diverse populations.

There are limitations to this study. First, the increased complexity of the model compared to other established prognostic scoring systems, namely MELD‐Na. Clinical information about the patient is required to complete the HELP scale. However, the clinical information is likely important and relevant to providers and patients that are contemplating EOL issues. In an effort to improve ease of use for providers, all clinical variables were made binary. The HELP scale is likely most useful in less timed constrained settings to aid in decision‐making regarding long‐term prognosis and advanced care planning.

Secondly, this study utilizes transplant‐eligible patients in this assessment of mortality from a data source not specifically designed to address hospice referral. Although this is a limitation because this patient population would be expected to be healthier than the general ESLD population, transplant eligibility is a variable construct and patients often shift in eligibility based on clinical and social circumstances. However, hospice referral provides an opportunity for addressing services and needs that is completely distinct from liver transplant eligibility. Patients, both transplant eligible and ineligible, may benefit from these services. Because hospice referral criteria only require a reasonable expectation of death in 6 months and major hepatic decompensation, there is a significant overlap with liver transplant listing criteria. This makes transplant wait list patients an ideal population to *begin* to explore a data‐driven approach and given the positive findings of our study, subsequently, explore its utility in expanded populations of transplant‐eligible and ineligible patients. The authors view the significance of the HELP scale as a novel prognostic indicator that provides an opportunity to facilitate shared decision‐making between patients and providers through largely objective means but with a more individualized approach than MELD‐Na, which remains largely underutilized. The aim of our prognostic scale is to facilitate increased comfort for patients and providers by utilizing hospice referrals earlier in the disease course. Generalizability will need to be examined in future studies in diverse ESLD patient populations.

## Conclusion

In conclusion, there has been a call to improve EOL prognostication in ESLD as our current strategies remain inadequate. The HELP scale can provide clinicians with a measure for identifying patients with limited life expectancy who may benefit from hospice services earlier than current referrals. Maximizing the use of hospice services may be an important step in improving the patient experience at the EOL for patients suffering from the complications of ESLD.

## Ethics statement

All listed authors meet the ICMJE definition of authorship.

## Data Availability

Data were obtained from the United Network for Organ Sharing (UNOS) STAR database utilizing publicly available, de‐identified data.
